# Do Carpets Impair Indoor Air Quality and Cause Adverse Health Outcomes: A Review

**DOI:** 10.3390/ijerph15020184

**Published:** 2018-01-23

**Authors:** Rune Becher, Johan Øvrevik, Per E. Schwarze, Steinar Nilsen, Jan K. Hongslo, Jan Vilhelm Bakke

**Affiliations:** 1 Domain of Infection Control and Environmental Health, Norwegian Institute of Public Health, P.O. Box 4404 Nydalen, N-0403 Oslo, Norway; Johan.Ovrevik@fhi.no (J.Ø.); PerE.Schwarze@fhi.no (P.E.S.); JanKenneth.Hongslo@fhi.no (J.K.H.); 2 SINTEF Building and Infrastructure, Pb 124 Blindern, 0314 Oslo, Norway; Steinar.Nilsen@sintef.no; 3 The Norwegian Labour Inspection Authority, P.O. Box 4720 Sluppen, 7468 Trondheim, Norway; Jan.Bakke@arbeidstilsynet.no

**Keywords:** carpets, indoor air quality, health impact

## Abstract

Several earlier studies have shown the presence of more dust and allergens in carpets compared with non-carpeted floors. At the same time, adverse effects of carpeted floors on perceived indoor air quality as well as worsening of symptoms in individuals with asthma and allergies were reported. Avoiding extensive carpet use in offices, schools, kindergartens and bedrooms has therefore been recommended by several health authorities. More recently, carpet producers have argued that former assessments were obsolete and that modern rugs are unproblematic, even for those with asthma and allergies. To investigate whether the recommendation to be cautious with the use of carpets is still valid, or whether there are new data supporting that carpet flooring do not present a problem for indoor air quality and health, we have reviewed the literature on this matter. We have not found updated peer reviewed evidence that carpeted floor is unproblematic for the indoor environment. On the contrary, also more recent data support that carpets may act as a repository for pollutants which may become resuspended upon activity in the carpeted area. Also, the use of carpets is still linked to perception of reduced indoor air quality as well as adverse health effects as previously reported. To our knowledge, there are no publications that report on deposition of pollutants and adverse health outcomes associated with modern rugs. However, due to the three-dimensional structure of carpets, any carpet will to some extent act like a sink. Thus, continued caution should still be exercised when considering the use of wall-to-wall carpeted floors in schools, kindergartens and offices, as well as in children’s bedrooms unless special needs indicate that carpets are preferable.

## 1. Introduction

Carpet floors in public buildings are used to reduce noise, especially in open plan offices and schools, but also for aesthetic reasons. During the 80s and 90s the use of such flooring in offices, schools, kindergartens and homes was shown to have negative effects on perceived indoor air quality. Carpets were also associated with adverse effects in users, particularly among those with asthma and allergy problems [1,2,3,4]. Later studies have supported an association between the use of carpets and adverse health outcomes. However, as far as we are aware of, the issue of carpets and health has not been reviewed before.

The carpet and rug producers have regularly argued that previous knowledge and risk assessments are obsolete and that modern rugs no longer represent a problem for indoor air quality. Rather, they now claim to represent a good option, even for people with asthma and allergies. From our own experience in Norway, we see that carpets are increasingly placed in new office buildings where the use of open plan offices are rising. Moreover, in our experience, the advice given earlier by health authorities to reduce the use of carpets in such premises is often ignored. Most likely, this is a trend that is common in many countries due to a potential cost reducing effect of open plan offices. That carpets are a common flooring material in large markets is reflected in the numbers reported on the home page of the Carpet and Rug Institute, which claims that carpets accounts for 51% of the total U.S. flooring market. Whether this number is on the rise or declining is not known to us.

In a health risk assessment of carpeted floor, two factors are of interest. One is that carpets may act as a repository for indoor air pollutants such as dirt, dust particles, allergens and other biological contamination that can build up in the carpets. Such pollutants may be processed, released and provide new exposure at a later time point. It should be emphasized that neither the presence of such pollutants in the carpet nor their resuspension is necessarily linked to health consequences unless the pollutants are hazardous and the exposure level is high enough to cause adverse effects.

The second is that carpets may emit volatile organic compounds (VOCs) that can cause smell and irritation of mucous membranes, especially in sensitive individuals. However, tests of new carpets indicate that emissions have been reduced and have a shorter duration. Pollution over a longer period depends partly on the deposition of dirt, care- and cleaning agents and on cleaning procedures [5]. To elucidate whether the recommendation to be cautious with the use of carpets is still valid, we reviewed the literature on the current knowledge on contaminants from carpets and adverse health outcomes associated with carpeted floors in non-industrial indoor environments. The review included literature from the late 80’s until today. Much of the older literature is still an important part of current knowledge. More recent publications support the old data in that carpets may act as a repository for pollutants that may become airborne during normal indoor activity and that adverse health outcomes could be associated with carpets.

## 2. Materials and Methods

A literature search in PubMed covering the years from 1980 to 2017 was performed. The search words in PubMed were:

Allergen levels, carpets, smooth floors: nine publications.
Carpets, adverse health effects: 149 publications.
Asthma, allergy, indoor environment, carpets: 61 publications.
Asthma and allergy associated with carpets: 59 publications.
Carpets, respiratory disease: 215 publications.
Flooring type, health impact: eight publications.

A review of the existing scientific literature (both epidemiological and experimental studies) including allergen levels/dust in carpets and on hard floors as well as possible adverse health effects associated with flooring type (carpets or other types) was performed. The first selection of possibly relevant publications was based on title and/or abstract. The different groups of search words retrieved mainly the same relevant publications. In addition we did a manual search on Google Scholar as well as on internet using the same search word used in PubMed. In total, 49 scientific publications were included. The studies consisted of measurements of pollutants (dust, allergens) in carpets compared with hard floors (for an overview, see Table 1) and cross-sectional, longitudinal and intervention studies (seven, four and four studies respectively) related to health impact. In addition there were one case control study, one in vitro experimental study and two studies looking at inflammatory markers in blood and genetic analysis respectively (see Table 2 for an overview of publications related to carpets and adverse health outcomes). As presented in this review, there are relatively few publications that have investigated the impact of carpeted floors on adverse health effects. Moreover, the majority of those that has investigated carpets and health have limited strength due to study size and design, with lack of objective health assessments. 

The publications that was retrieved by our search terms but excluded from the review where typically dealing with industrial indoor environments, occupational exposure (such as exposure of textile workers) or did not present relevant data on carpets and health.

## 3. Results

### 3.1. The Carpet as a Possible Source of Exposure

A number of studies have shown significantly higher levels of dust as well as allergens from fungi, dog, cat and house dust mite in carpets compared to smooth/hard floors [6,7,8,9,10,11,12]. Accordingly, Matheson et al. [13] found that installing carpets caused an increased exposure to allergens from house dust mites. Removing the carpets significantly reduced the levels of both mite allergens and ergosterol, a component of the cell wall of molds. Similar results were reported in a study comparing environmental characteristics of carpets and hard floors, where significantly larger amount of dust deposits and higher percentage of viable microorganisms were found in the carpet floor compared to the hard floors [5].

In a later review evaluating contributing factors to indoor allergen exposures in schools and kindergartens and the impact on asthma and allergy disease, carpeted floors, upholstered furniture and clothes were important reservoirs and sources of allergens, especially from dust mites and pets [14]. When comparing the recovery fraction obtained by vacuuming of standardized dust applied to various surfaces, significantly lower amounts were obtained from rough and porous surfaces compared with smooth and hard surfaces and with the lowest recovery from carpets [15]. This indicated that dust could be collecting in the carpet, but whether this is more easily suspended later was unclear.

A study in Belgian schools found that the ratio between the amounts of particulate matter with aerodynamic diameter less than 2.5 µm (PM_2.5_) in indoor and outdoor air was significantly higher for classrooms with carpeted floors compared with classroom without carpet floor. This indicated that carpets may increase the amount of resuspended (swirled) dust [16]. Tian and colleagues [17] examined how simulated walk on the floor lead to resuspension of dust and how this is affected by factors such as type of floor. For particles in the size fraction from 0.4 to 3.0 µm the differences in amounts stirred up were not significantly different between carpeted and hardwood floors. For particles in size fraction between 3.0 to 10.0 µm, more particles were resuspended from carpeted floors compared to hard floors. This indicates (depending on particle size) that the higher dust amounts reported for the carpeted floors are likely to come from increases in the coarse fraction.

Similar results were shown in chamber studies by Bramwell and coworkers [18] who found that flooring type can significantly impact incremental time-averaged daily exposures to coarse and fine particles and that high-density cut pile carpeting resulted in the highest exposures. Overall, the authors found that resuspension from walking within the residential micro-environment contributed between 6–72% of time-averaged daily exposures to PM_10_. Similar results were obtained by Patton and coworkers regarding pathogenic microorganisms [19]. By using an environmental chamber, they quantified re-aerosolization caused by walking on either industrial carpet or polyvinyl chloride (PVC) floor coverings contaminated with bacterial spores. The results showed that walking on a carpet generated significantly more re-aerosolization of spores than walking on PVC. Heavy contra light activity as well as the height above the floor where the sampling was done had an impact on the amount of re-aerosolization levels [19].

Higher resuspension rates of particles from carpets compared to hard floor materials were also found in the chamber study by Siming and Wan [20]. For both carpet and wood polyvinyl chloride (PVC), the resuspension rate was higher for PM_10_ than for PM_2.5_. Independent of surface material type, increased humidity resulted in decreased resuspension rates.

In agreement with these findings are the results from a more recent chamber study [21] where the impact of humidity and air swirl on resuspension of various biological particles from different surface materials were investigated. They found that particle resuspension rate was dependent on particle size, the hydrophilic properties of the particles and relative humidity (RH). Thus, resuspension of hydrophilic particles (house dust mites) but not hydrophobic particles decreased when RH was increased and more so from linoleum compared to carpets. However, the resuspension of the hydrophilic particles was higher from carpets than linoleum at all three levels of investigated RH (42%, 63% and 82%).

In contrast, there are also studies indicating that deposited dust in carpets not easily become airborne. In the study by Dahl et al. [5], the significantly larger amount of dust deposits and higher percentage of viable microorganisms found in the carpet floor as compared to the hard floors did not result in observed differences in dust fall rates on inventory surfaces or particle content in room air.

Another study, investigated the dust reducing effect of new carpets and air purification combined in classrooms. The combination resulted in lower PM_10_, PM_2.5_ and VOC levels in air than for hard floors alone [22]. However, since this study examined only the combination of carpet and air purifiers, the result cannot be used to evaluate the contribution of carpets only.

Carpet suppliers have claimed that carpets constructed with straight fibers, i.e., they do not form the shift or loop of more traditional textile carpets, gathers less dirt and is easier to keep clean. However, significantly higher concentrations of allergens from house dust mites have been reported in rooms with carpet floors no matter how the carpets were made (loops or straight/angled fibers), compared to hard/smooth floors [10]. It also appears that the amounts of allergens on carpet floors were highest in the deepest layers of the carpets and that the rug structure had little impact on how much allergen that could be detected [10].

Overall, the available literature indicates that carpet flooring acts as a reservoir for accumulation of dust/indoor contaminants and microbiological material. It also appears that carpeted floors also contribute to increased amounts of suspended dust and microorganisms in indoor air but this may depend on relative humidity, particle size and hydrophilic properties of the particles. Further studies are required to conclude on this matter. However, we find no clear evidence in peer-reviewed journals that carpet flooring in general has dust-reducing capacity.

### 3.2. Carpets and Adverse Health Outcomes

Over the years, several studies have investigated the impact of indoor environmental factors on health. Different adverse health outcomes were found to be associated with exposure to carpets.

Early longitudinal studies in the 80s and 90s found that staff in schools with carpet floors reported more eye irritation, swollen eyelids, and nasal and respiratory irritation (dry/sore throat, irritation cough), facial rash, headache, abnormal fatigue and feeling of being cold [1,2]. The proportion of people who reported on static electricity was also higher for carpeted floors. Removal of the carpets reduced the symptoms of eye and nose irritation, headaches and fatigue, to a level corresponding to carpet-free premises. The studies found no relationship between carpet floors and coryza/rhinitis, nasal congestion, nausea, itching (face/hands) rash on the hands or eczema. Neither were all symptoms (throat irritation, coughing and feeling of being cold) relieved upon intervention. Since the carpets were 8–10 years old and no chemical cleaning agents had been used, the authors considered it unlikely that VOCs from carpets or cleaning products were the reason for the experienced health problems. Rather, the study indicates the importance of dust and allergens.

Another “early” study finding an association between carpets and adverse health effects was the cross sectional “Danish Town Hall Study” which investigated the impact of various indoor air parameters on the incidence of mucosal irritation as well as more general symptoms such as fatigue and headaches among employees [3]. Quantities of floor dust and type of flooring were two of several parameters associated with the occurrence of symptoms. A statistically significant association was found between mucosal irritation (irritation/dryness of the eye nose and throat) and the use of carpets with an odds ratio (OR) > 1.5 for felt carpets (more than 1.5 times increased frequency of the disease outcome) and OR > 2.0 for loop-woven rugs. Later, two studies from Sweden and Denmark using climate chambers and with the same study protocol, observed positive effects on symptoms such as mucosal irritation, irritation of eyes, dry skin, perceived indoor air quality and the quality and speed of execution of tasks after removal of an old carpet floor [23,24,25]. Since these studies were performed in climate chambers in which possible confounding causes for this type of symptoms were controllable, the results support previous epidemiological findings describing associations between carpets and irritative symptoms, mild cognitive effects and perceived air quality.

In the more recent European OFFICAIR study, a cross-sectional study among 7441 office employees in 167 “modern” European office buildings (new or retrofitted, preferably >10 years old, surveyed between 2011 and 2012), building associated symptoms were evaluated by a so-called Building Symptom Index (BSI) which is based on five symptoms: dry eyes, nasal congestion, dry/irritated throat, headache and lethargy (fatigue) [26]. A significant increase in BSI was observed in buildings where carpeting was the most commonly used flooring material in the offices. Considering that the majority of carpeted floors in these buildings likely were installed after 2001–2002, this suggest that even more recent carpet materials could represent an indoor air problem.

With respect to more serious adverse health outcomes, a case-control study by Jaakkola and coworkers [27] found indications of an increased risk of asthma among adults associated with wall-to-wall carpets in the workplace, but the effect was not statistically significant. Carpet floors in the homes were not associated with asthma. Nor were molds significantly associated with asthma. But the combination of carpeted floors and the presence of mold in the workplace gave a significantly increased risk of asthma (OR 4.64). An increase in risk for asthma by combination of carpeted floors and mold in the home was also observed, but this was not statistically significant [27].

An association between asthma and carpets was also found in a case-control study among 579 children (12–14 years; 193 with asthma and 386 healthy controls) in Taiwan [28]. The authors observed that carpets placed before the child was 5 years were associated with an increased risk of childhood asthma, and in particular the risk of early asthma development. Carpets in other bedrooms were not significantly associated with childhood asthma. Also the result of Ekici and coworkers [29] emphasize the importance of children’s early exposures to environmental factors including carpets for disease outcome. They found that the presence wall to wall carpets in the home during childhood were one of several factors that contributed to frequent respiratory infections in childhood as well as asthma at adult age.

An association between carpets and early onset asthma was also observed in the study by Ferry and coworkers [30] where they looked to identify environmental exposures associated with early onset of asthma and potential hereditary impact. More than 1000 individuals with physician diagnosed asthma and disease onset by two years or older were studied. The study included information about the children’s early exposure to 17 environmental factors. The presence of singly or combinations of these early exposures were tested for association with variation in the age at onset asthma. For exposures that could be linked to the age at onset, the study tested whether the effect could be modified by genetic variants where an association with allergic disease was known. Carpeted home was among five environmental exposures that could be linked to variation in age at asthma onset. The other exposures included serious respiratory illness and direct exposure to the father’s smoking.

For individuals with early-onset asthma (between two and six years) there was an increased likelihood that they had lived in a house with carpets (OR 1.4) and that they reported a serious respiratory illness before the age of two compared with individuals with later asthma onset (OR 2.1). The risk of an early asthma onset was further increased if carpet exposure and severe respiratory disease both occurred before two years of age (OR 3.2). No significant interactions between gene variants and environmental exposure at the age of asthma onset were observed. The authors state that these findings indicate that age of asthma onset in individuals at high risk of developing the disease may be delayed by avoiding carpet exposure and preventing severe respiratory disease during the first two years of life.

Also an Australian case-control study found that carpets in the child’s bedroom gave significantly higher risk of re-hospitalization due to asthma attacks (OR 4.07) compared with the overall incidence of carpeting in the home, which showed no significant risk [31]. In a French birth cohort of 1879 children (less than 18 months), both mild and severe wheezing was associated with carpet floor (OR 1.39) [32]. A large cross-sectional study among 23,326 Chinese children also reported that carpeted floors in the bedroom were significantly associated with increased risk of (self-reported) asthma (OR 1.94), wheezing (OR 1.47) and chronic cough (OR 1.40) [33].

Taken together, the available data suggest that indoor air quality and the presence of carpets especially in the child’s bedroom is important for preventing the development or exacerbation of childhood asthma as well as asthma at adult age.

Interleukin (IL)-13 is an inflammatory cytokine involved in allergic responses. Genetic variation (genetic polymorphism) in the IL-13 gene has been shown to affect health outcomes in the airways. A recent study looked at the impact of variations in IL-13 gene has on asthma phenotypes (variants of asthma disease with different characteristics) in a group of 3577 Taiwanese children [34]. Information about children’s exposure and disease status was obtained through a questionnaire completed by the children’s parents. For children with a particular variant (haplotype) of IL-13 gene, there was an increased risk of wheezing (OR 1.5). The risk of wheezing and asthma (after five years) increased significantly if children with this gene variant lived in a carpeted home (OR respectively 2.5 and 4.7). This indicates that variations in the IL-13 gene could be linked to asthma disease in children and that asthma disease was related to carpet use. These results are interesting, but must be confirmed in other similar studies.

Some studies have shown current use of carpets to be associated with a reduced prevalence of asthma [35,36,37,38]. This has been suggested to indicate either a true positive effect or a misinterpretation (bias) caused by a more frequent use of carpets among those who have children without increased risk of asthma. A possible reason for the latter is extensive advising since the 70’s to patients with asthma to avoid carpets and dust mites. This has probably resulted in less carpet use among these compared to those without asthma (selection bias) [4]. Zock, Behrens as well as Skorge and Mommers [35,36,37,38] concluded that such bias was a likely cause of their findings. This is particularly a problem in cross-sectional studies of overall asthma prevalence. Case-control studies and longitudinal studies, and studies of new-diagnosed asthma and frequency of asthma symptoms are likely to be less affected by such bias, depending on the availability of information on confounders.

Also other findings have indicated lack of adverse health impact from carpets. A study 400 individuals with uncontrolled asthma found no impact from the carpet floor in the bedroom [39]. However, the findings had a limited predictive power because of the low percentage that had carpeted floors in the study (about 12%).

Voûte and coworkers [40] tested whether wall-to-wall carpets in schools had a negative impact on the respiratory health of asthmatic children. They found no significant difference between children visiting schools with or without carpeted classroom floors in peak-flow variability, acute respiratory symptoms, or medication use. However, the house dust mite allergen content in dust collected from classroom floors was much lower than of dust collected from homes. The authors concluded that the lack of contribution from carpeted classroom to asthma symptom severity could be due the low levels of mite allergens on them.

Some studies also points to newly installed carpet flooring as a cause of various health outcomes. In a cross-sectional survey among 5951 Russian children between 8 and 12 years, wheezing (sustained periods of dry cough, tightness or wheezing) and allergy were associated with new synthetic carpets and other new synthetic flooring installed during the last 12 months [41]. Correlation with asthma was also observed but was not statistically significant. Associations with the same health outcomes were significantly lower if the carpets were older than 12 months. This study design indicate that emissions from new flooring materials in general during the first 12 months, such as VOCs, and not deposited dust, allergens or microorganisms, were the most likely cause of the observed health outcomes.

Also other results suggest that emissions from materials used in indoor environments have an impact on adverse health outcomes. Shu and coworkers found that exposure to PVC flooring material in early life was associated with incidence of asthma at a later time when compared with other flooring materials (not including carpets specifically) and especially when comparing with wood flooring type [45]. It is speculated that this could be caused by exposure to phthalates from the PVC flooring material.

In relation to indoor home renovation, inflammation markers in blood from 250 six year old children were analyzed [42]. Information on paint, flooring and new furniture was provided by the children’s parents. Elevated levels of inflammatory cytokines were associated with refurbishment activities. Among the flooring types only newly installed carpets were significantly associated with elevated blood concentrations of some inflammatory mediators in the children. More recent studies also indicate that carpets may impact on a number of symptoms. In 2009, a bank underwent a complete renovation which included installation of new carpets [43]. After starting to use the premises, high numbers of workers developed symptoms such as eczema, coryza and hives (urticaria). After removal of the carpets, the symptoms diminished in the same individuals. Isothiazolinone and fumarates both of which can provide respiratory and skin symptoms were isolated from carpets but the study could not determine that these compounds were the cause of the symptoms.

Influenza A and B viruses have been shown to survive on solid, non-porous surfaces 24–48 h and the transfer from such surfaces to the hands is possible [46]. On materials like textiles and paper, virus survival time was shorter (less than 8–12 h). Transmission of the virus from smooth surfaces to hands could occur for 24 h and from textiles/fabrics for 15 min. Similar results have also been demonstrated for other types of viruses which survived longer on smooth surfaces [47]. Whether this has any practical significance regarding the use of carpets are unclear. Carpets can also possibly function as a reservoir for virus particles [48]. At a hospital in which two persons removed a carpet floor 13 days after the last case of an outbreak of Norovirus, both became ill. Daily vacuuming after the outbreak had apparently not cleared the virus. However, there is a possibility that surviving viruses could have occurred on any surface in the room and that workers were exposed due to the time that they spent in the room. This indicates how complex exposure can be with respect to virus particles and flooring. A potential impact of infection will likely depend on several factors such as surface materials, air humidity, temperature and type of virus.

Overall, the literature suggests that the use of carpets is associated with an increased risk of adverse health outcomes in the form of mucosal irritation, fatigue, asthma and inflammatory responses. Of notice, the majority of these observations are based on epidemiological studies. However, results from an in vitro study provide some experimental support for this notion. Floor dusts from 12 rooms in two schools were quantified and examined for inflammation promoting properties in a lung epithelial cell line. Not only were the dust levels significantly higher on carpeted floors, but the carpet-dust also had a higher inflammation inducing capacity than dust from smooth floors [44].

## 4. Discussion

The available literature shows that carpets may act as a repository/sink for indoor air pollutants such as particles, allergens and other biological contamination (see Table 1). Worth considering is that carpets provide particular challenges in relation to cleaning [5,49], requiring much more comprehensive cleaning procedures with higher financial costs compared to hard floors. In contrast to hard and semi-hard coverings, carpets can hide dirt and dust that do not form visible aggregates. With the purpose to save maintenance and cleaning costs, there is a risk that proper carpet cleaning are postponed and not performed regularly as recommended. This could increase the accumulating levels of contaminants in the carpet.

Some studies have also investigated whether these pollutants are released back into the indoor environment. Most, but not all of these studies indicate that carpet flooring leads to increased amounts of such pollutants in indoor air compared to smooth floors, most likely through resuspension of deposited material [5,17,18,19,20]. Recent data indicates that for some types of such deposited material, the potential for resuspension depends on the relative humidity and hydrophobic properties of the material as well as particle size [21]. Furthermore, and most importantly, the peer-review literature provides no clear evidence for a dust-reducing effect of carpet flooring.

The majority of studies appear to find correlation between carpet floors and adverse health outcomes such as respiratory infections, asthma worsening and age at asthma onset [27,29,30,31,34]. Carpets in the children’s bedroom have also been associated with increased risk of asthma [31,33] However, the current knowledge is insufficient to quantify the risk for these outcomes. This is the same for more diffuse health problems such as headaches, mucosal irritation and fatigue.

Although the identified studies can be used for an overall evaluation, direct comparisons of the reported results are challenging since the type of carpets included in the studies (i.e., wall-to-wall carpeting or loose carpets) rarely is specified. Although it is it is reasonable to assume that studies on offices/workplaces mostly involves wall-to-wall carpeted floors, there information about the rug-design/construction, cleaning procedures, type of maintenance, age of carpet as well as ventilation rates tend to be lacking. These are all factors that could potentially influence on pollutant levels and adverse health impact.

That being said, it has not been clearly shown which factors are associated with carpet floors that could be the cause of the reported health effects. Dust, allergens and VOC are the most obvious candidates, but the available literature provides no unambiguous clarification regarding this. When choosing materials to replace carpets, a thorough assessment of possible effects from the alternatives on indoor air quality and health should be performed. The same applies to “fleecy” wall and roofing materials, which should be considered similar to carpets when selecting silencing materials.

Carpeted floors as well as most other flooring types may also emit VOCs that can smell and irritate the mucous membranes in susceptible individuals. However, the carpet industry claims that emissions from new carpets are low and last for a short time. Independent of initial levels, VOC-emissions from the carpet itself or glue products used during carpeting will be reduced over time. Hence, VOC-release from old carpeted floors is more likely due to emission of substances supplied to the carpet, such as cleaning products. Notably, emission problems related to new flooring is not restricted to carpets, and may also occur with newly installed hard-floor materials [41].

Onset or exacerbation of asthma is affected by a variety of risk factors including several that can be found in the indoor environment. To reduce the burden of disease, avoidance of widespread use of carpets should be among factors considered. For this, reason use of carpeted floors in schools and kindergartens should also be avoided, at least until more scientific knowledge is provided.

## 5. Conclusions

There is still a need for more knowledge about the possible health impacts of carpet flooring, especially from new types of carpeted floors that have come on the market in recent years. This will require cohort studies with larger study populations and interventions as well as doctor diagnosed disease outcomes. Supplemental in vitro studies can be valuable to add plausibility to findings in epidemiological studies.

So far, we have not found peer-reviewed evidence supporting the notion that modern carpets now are unproblematic for the indoor environment. On the contrary, the literature suggests that the use of carpets is linked to increased levels of indoor dusts, allergens, and microorganisms, and associated with increased risk of a number of health outcomes including mild cognitive effects, irritative symptoms, and asthma. Caution should therefore still be exercised when using carpeted floors in homes, schools, kindergartens and offices unless special needs make carpets preferable. Acoustics problems can in many cases be solved in other ways than by using carpet flooring.

## Figures and Tables

**Table 1:** Carpets as an exposure source.

Authors [Reference]	Type of Study	Results
Dybendal et al. [6]	Comparison of dust, proteins and allergens from carpeted and smooth floors in homes and schools in Norway.	Carpeted floors in schools and homes contained significantly more dust, proteins, and allergens than smooth floors.
Van Strien et al. [7]	Measurements of mite allergen concentrations in floor- and mattress dust in dwellings.	Mite allergen concentrations in dust from carpeted floors were 6–14 times higher than in dust from smooth floors.
Zock et al. [8]	Comparison of house dust mite allergen levels in dust from schools with smooth and carpeted classroom floors.	More house dust mite allergen in dust from carpeted classroom floors than from smooth classroom floors, but the levels were much lower than in dust from floors in homes.
Tranter [9]	Review article.	Higher concentrations of allergens consistently found in carpets compared with smooth floors. Carpet vacuuming seems to remove larger particles but not the allergen-associated smaller particles whereas smooth floor cleaning appears more efficient regarding removal of these smaller particles.
Causer et al. [10]	Effect of carpet construction on content and vertical distribution of mite allergen.	Significantly higher concentrations of allergens from house dust mites have been reported in rooms with carpet floors no matter how the carpets were made compared to hard/smooth floors.
Arbes et al. [11]	Levels of seven indoor allergens in 89 day-care facilities in two North Carolina counties. Dust samples were collected from carpeted and non-carpeted areas of one room each place.	Levels of several antigens were statistically higher on carpet than on hard surfaced flooring. For dog and cat allergens, the differences were clinically significant, with mean levels on hard floors being well below proposed thresholds for allergic sensitization.
Cho et al. [12]	The effect of home characteristics on dust antigen concentrations in homes.	Carpeted floor held larger amount of antigens than non-carpeted floor.
Matheson et al. [13]	Residential characteristics predict changes in Der p 1, Fel d 1 and ergosterol but not fungi over time.	Installation of carpets caused an increased exposure to allergens from house dust mites. Removal of carpets significantly reduced the levels of both mite allergens and ergosterol, a cell wall component of molds.
Dahl et al. [5]	Textile floor coverings as part of indoor environment.	Significantly larger amount of dust and viable microorganisms in carpet floors compared to hard floors did not result in observed differences in dust fall rates on inventory surfaces or particle content in room air.
Salo et al. [14]	Investigation of indoor allergens in school and day care environments.	Carpeted floors, upholstered furniture and clothes were important reservoirs and sources of allergens, especially from dust mites and pets.
Ashley et al. [15]	Evaluation of a standardized micro-vacuum sampling method for collection of surface dust.	When comparing the recovery fraction obtained by vacuuming of standardized dust applied to various surfaces, significantly lower amounts were obtained from rough and porous surfaces compared with smooth and hard surfaces and with the lowest recovery from carpets.
Stranger et al. [16]	Comparative study of indoor air quality in Belgian schools.	The ratio between indoor air and outdoor air amounts of particulate matter (<2.5 mm) was significantly higher for classrooms with carpeted floors compared with classroom without carpet floor. This indicated that carpets may increase the amount of resuspended dust.
Tian et al. [17]	A comparative study of walking-induced dust resuspension using a consistent test mechanism.	For particle size 3.0–10.0 µm, carpets exhibited higher resuspension fractions compared with hard floorings. The results support that people sensitive to allergens could select hard floorings to reduce exposure and adverse health outcomes.
Bramwell et al. [18]	An evaluation of the impact of flooring types on exposures to fine and coarse particles.	Flooring type can significantly impact incremental time-averaged daily exposures to coarse and fine particles and that high-density cut pile carpeting resulted in the highest exposures.
Paton et al. [19]	Study of re-aerosolization of spores from flooring surfaces.	Walking on a carpet generated significantly more re-aerosolization of spores than walking on PVC. Heavy contra light walking as well as height above the flooring material where sampling was done all had an impact on the amount of re-aerosolization levels.
Siming et al. [20]	Chamber study.	Higher resuspension rates of particles from carpets compared to wood PVC and vinyl materials.
Salimifard et al. [21]	Chamber study.	Carpet surfaces yields higher resuspension rate than linoleum surface at the same humidity level.
Scheepers et al. [22]	The effect on indoor air quality of new carpet combined with air filtration investigated in classrooms.	The combination resulted in reduced levels of particles and VOC in air compared with hard floors alone. No assessment was done on carpets alone.

**Table 2:** Associations between carpet use and adverse health outcomes.

Authors [Reference]	Type of Study	Results
Norbäck et al. [1].	Longitudinal. Questionnaire. (Initial cross-sectional study with self-reporting of symptoms followed by later questionnaire).	Personnel in schools with wall-to-wall carpet reported increased prevalence of eye and airway symptoms, face rashes, headache and abnormal tiredness compared with those in schools with hard floors. Removal of carpets caused several symptoms to decrease. Frequency of airway symptoms remained increased in the carpet group.
Norbäck et al. [2]	Longitudinal study. A four year study among personnel in six primary schools.	Chronic SBS was related to VOC, previous wall to wall carpeting in the schools, hyperreactivity, and psychosocial factors.
Skov et al. [3]	Cross sectional (self-reported). 2369 office workers in 14 buildings, where indoor climate measurements were made, filled out a questionnaire.	Floor covering, the shelf factor and the fleece factor were among several factors associated with the prevalence of symptoms (work-related mucosal irritation and work related general symptoms).
Wargocki et al. [23]	Reversible intervention study. Experimental study with cross-over design. Persons unaware of intervention were exposed once with the pollution source present and once without the pollution source. Self-reporting of symptoms and evaluation of task performance.	Pollution source was a 20 years old carpet. Removal of pollution source resulted in increased satisfaction with perceived indoor air, reduced prevalence of headaches and significantly faster typing of text. Reducing the pollution load was effective in improving comfort, health and productivity.
Wargocki et al. [24]	Reversible intervention study. Experimental study with a cross-over design. Persons unaware of the intervention. Self-reporting of symptoms and evaluation of task performance.	Carpets used as pollution source. Overall productivity increased with increased ventilation. Results show that maintaining good indoor air quality by controlling indoor pollution sources and ensuring adequate ventilation important for comfort, health and productivity.
Wargocki et al. [25]	Reversible intervention study. Subjective assessments of perceived air quality, intensity of sick building syndrome symptoms and performance of office work.	Removing the pollution source improved the perceived air quality, decreased the perceived dryness of air and the severity of headaches, and increased typing performance.
Bluyssen et al. [26]	Self-reported (Cross sectional study).	An increase in adverse health effects was observed in offices where carpet was the main type of floor covering.
Jaakkola et al. [27]	Cross sectional. (Population-based incident case-control study)	The risk of asthma was related to the presence of plastic wall materials and wall-to-wall carpet at work, the latter in particular in the presence of mold problems (adjusted OR = 4.64, 95% CI: 1.11, 19.4).
Chen et al. [28]	A 1:2 matched case-control study. Self-reported.	Cockroaches, carpet, pets, and in-utero exposures to ETS affected the timing of early-onset asthma.
Ekici et al. [29]	Retrospectiv cross sectional. Parents and grandparents of schoolchildren were asked to answer questionnaires about respiratory system-related symptoms and characteristics in the children.	Childhood respiratory infections associated with increased risk of asthma, chronic bronchitis and chronic cough. Several factors including wall-to-wall carpets were associated with increased risk of frequent childhood respiratory infections.
Ferry et al. [30]	Longitudinal. Exposures to 17 environmental factors before the age of two were reported retrospectively.	Individuals with early childhood asthma more likely to have lived in a house with carpet and more likely to report suffering a serious chest illness before the age of two compared to those with later asthma onset. Carpet exposure and suffering a serious chest illness concurrently before age two increased the individual risk even more.
Vicendese et al. [31]	Hospital-based case-control study.	Carpeted floors in the bedroom associated with increase in asthma readmissions (OR = 4.07, 95% CI 1.03–16.06).
Herr et al. [32]	Longitudinal (Birth cohort). Data on wheezing disorders, medical visits and medications, as well as biological markers of atopy, were collected at 18 months.	Prevalence of wheeze was influenced by several factors where carpet covered floor in the child’s bedroom was one.
Liu et al. [33]	Cross-sectional study.	Bedroom carpets were one of several indoor factors associated with higher prevalence of respiratory symptoms.
Tsai et al. [34]	Genetic analysis and questionnaire about children’s exposure and disease status.	Genetic variation in the IL-13 gene affects health outcomes in the airways. This study indicated that variations in the IL-13 gene could be linked to asthma in children and that asthma disease was related to carpet use.
Zock et al. [35]	Housing data obtained by an interviewer-led questionnaire. Associations between housing data and asthma (based on symptoms the last year) as well as bronchial responsiveness were evaluated.	Carpets and rugs in the bedroom were related to less bronchial responsiveness and fewer asthma symptoms.
Behrens et al. [36]	Data from two cross sectional surveys 5 years apart.	Carpets were inversely associated with adverse respiratory conditions but not when the analysis was restricted to individuals with no report on carpet avoidance due to a history of atopic disease.
Skorge et al. [37]	Self-reported (Cross sectional study).	Wall to wall carpets in the bedroom was negatively associated with cough with phlegm, chronic cough, and attacks of dyspnoea.
Mommers et al. [38]	Nested case-control study. Part of a longitudinal study in children on respiratory health and indoor environment.	A negative association between asthma symptoms and wall-to-wall carpets was observed
Al-Zahrani et al. [39]	Cross sectional study	Carpets in the bedroom were among several factors that did not appear related to the extent of asthma control.
Voute et al. [40]	Longitudinal. Participating children had peak-flow measurements three times a day for a 1-month period. Concurrently, the parents recorded respiratory symptoms and medication use daily.	Peak-flow variability in asthmatic children not related to wall-to-wall carpeting on classroom floors.
Jaakkola et al. [41]	Cross sectional.	Risk of current asthma, wheezing, and allergy in Russian children were related to recent renovation and the installation of materials with potential chemical emissions. This included new linoleum flooring and synthetic carpeting, particleboard which were determinants of one or several of the adverse health outcomes.
Herberth et al. [42]	Blood sampling, inflammatory markers. Within a birth cohort study, blood samples of 6 year old children were analysed for concentration of inflammatory markers.	Increased levels of inflammatory markers were related to renovation activities, in particular, new floor covering. Among floor covering materials only wall-to-wall carpets were associated with elevated IL-8 and Monocyte Chemoattractant Protein-1 (MCP-1) levels.
Ebbehøj et al. [43]	Intervention, cohort.	Workers developed skin and/or airway problems after renewal of offices (new furniture and carpets). Removal of carpets significantly improved symptoms. Workers were examined in 2009 and re-examined in 2013. Chemicals from glued carpets suspected as trigger.
Allermann et al. [44]	In vitro study. The inflammatory potency of floor dust was measured as interleukin-8 secretion from the lung epithelial cell line A549 after exposure to dust.	Carpet flooring may act as a “sink” for microorganisms resulting in a higher inflammatory potency of floor dust

## References

[1] Norbäck D., Torgén M. (1989). A longitudinal study relating carpeting with sick building syndrome. Environ. Int..

[2] Norbäck D., Torgén M., Edling C. (1990). Volatile organic compounds, respirable dust, and personality factors related to prevalence and incidence of sick building syndrome in primary schools. Br. J. Ind. Med..

[3] Skov P., Valbjørn O., Pedersen B.V. (1990). Influence of indoor climate on the sick building syndrome in an office environment. Scand. J. Work Environ. Health.

[4] Institute of Medicine (IOM) (2000). Clearing the Air. Asthma and Indoor Exposures.

[5] Dahl I.E., Holøs S.B., Nilsen S.K. Textile floor coverings as part of indoor environment. I: Levin, Hal, Gina Bendy and Joyce Cordell (red). Proceedings of the 9th International Conference on Indoor Air Quality and Climate.

[6] Dybendal T., Elsayed S. (1994). Dust from carpeted and smooth floors. VI. Allergens in homes compared with those in schools in Norway. Allergy.

[7] Van Strien R.T., Verhoeff A.P., Brunekreef B., Van Wijnen J.H. (1994). Mite antigen in house dust: Relationship with different housing characteristics in The Netherlands. Clin. Exp. Allergy.

[8] Zock J.P., Brunekreef B. (1995). House dust mite allergen levels in dust from schools with smooth and carpeted classroom floors. Clin. Exp. Allergy.

[9] Tranter D.C. (2005). Indoor allergens in settled school dust: A review of findings and significant factors. Clin. Exp. Allergy.

[10] Causer S., Shorter C., Sercombe J. (2006). Effect of floorcovering construction on content and vertical distribution of house dust mite allergen, Der p 1. J. Occup. Environ. Hyg..

[11] Arbes S.J., Sever M., Mehta J., Collette N., Thomas B., Zeldin D.C. (2005). Exposure to indoor allergens in day-care facilities: Results from 2 North Carolina counties. J. Allergy Clin. Immunol..

[12] Cho S.H., Reponen T., Bernstein D.I., Olds R., Levin L., Liu X., Wilson K., Lemasters G. (2006). The effect of home characteristics on dust antigen concentrations and loads in homes. Sci. Total Environ..

[13] Matheson M.C., Dharmage S.C., Forbes A.B., Raven J.M., Woods R.K., Thien F.C., Guest D.I., Rolland J.M., Haydn Walters E., Abramson M.J. (2003). Residential characteristics predict changes in Der p 1, Fel d 1 and ergosterol but not fungi over time. Clin. Exp. Allergy.

[14] Salo P.M., Sever M.L., Zeldin D.C. (2009). Indoor allergens in school and day care environments. Allergy Clin. Immunol..

[15] Ashley K., Applegate G.T., Wise T.J., Fernback J.E., Goldcamp M.J. (2007). Evaluation of a standardized micro-vacuum sampling method for collection of surface dust. J. Occup. Environ. Hyg..

[16] Stranger M., Potgieter-Vermaak S.S., Van Grieken R. (2007). Comparative overview of indoor air quality in Antwerp, Belgium. Environ. Int..

[17] Tian Y., Sul K., Qian J., Mondal S., Ferro A.R. (2014). A comparative study of walking-induced dust resuspension using a consistent test mechanism. Indoor Air.

[18] Bramwell L., Qian J., Howard-Reed C., Mondal S., Ferro A.R. (2016). An evaluation of the impact of flooring types on exposures to fine and coarse particles within the residential micro-environment using CONTAM. J. Expo. Sci. Environ. Epidemiol..

[19] Paton S., Thompson K.-A., Parks S.R., Bennett A.M. (2015). Reaerosolization of spores from flooring surfaces to assess the risk of dissemination and transmission of infections. Appl. Environ. Microbiol..

[20] Siming Y., Man P.-W. (2015). Experimental investigation and modelling of human-walking-induced particle resuspension. Indoor Built Environ..

[21] Salimifard P., Rim D., Gomes C., Kremer P., Freihaut J.D. (2017). Resuspension of biological particles from indoor surfaces: Effects of humidity and air swirl. Sci. Total Environ..

[22] Scheepers P.T., de Hartog J.J., Reijnaerts J., Beckmann G., Anzion R., Poels K., Godderis L. (2015). Influence of combined dust reducing carpet and compact air filtration unit on the indoor air quality of a classroom. Environ. Sci. Process. Impacts.

[23] Wargocki P., Wyon D.P., Baik Y.K., Clausen G., Fanger P.O. (1999). Perceived air quality, sick building syndrome (SBS) symptoms and productivity in an office with two different pollution loads. Indoor Air.

[24] Wargocki P., Wyon D., Sundell J., Clausen G., Fanger P.O. (2000). The Effects of outdoor air supply rate in an office on perceived Air quality, sick building syndrome (SBS) symptoms and productivity. Indoor Air.

[25] Wargocki P., Lagercrantz L., Witterseh T., Sundell J., Wyon D.P., Fanger P.O. (2002). Subjective perceptions, symptom intensity and performance: A comparison of two independent studies, both changing similarly the pollution load in an office. Indoor Air.

[26] Bluyssen P.M., Roda C., Mandin C., Fossati S., Carrer P., de Kluizenaar Y., Mihucz V.G., de Oliveira F.E., Bartzis J. (2016). Self-reported health and comfort in ‘modern’ office buildings: First results from the European OFFICAIR study. Indoor Air.

[27] Jaakkola J.J., Ieromnimon A., Jaakkola M.S. (2006). Interior surface materials and asthma in adults: A population-based incident case-control study. Am. J. Epidemiol..

[28] Chen Y.C., Tsai C.H., Lee Y.L. (2011). Early-life indoor environmental exposures increase the risk of childhood asthma. Int. J. Hyg. Environ. Health.

[29] Ekici M., Ekici A., Akin A., Altinkaya V., Bulcun E. (2008). Chronic airway diseases in adult life and childhood infections. Respiration.

[30] Ferry O.R., Duffy D.L., Ferreira M.A.R. (2014). Early life environmental predictors of asthma age-of-onset. Immun. Inflamm. Dis..

[31] Vicendese D., Dharmage S.C., Tang M.L.K., Olenko A., Allen K.J., Abramson M.J., Erbas B. (2015). Bedroom air quality and vacuuming frequency are associated with repeat child asthma hospital admissions. J. Asthma.

[32] Herr M., Just J., Nikasinovic L., Foucault C., Le Marec A.M., Giordanella J.P., Momas J.I. (2012). Influence of host and environmental factors on wheezing severity in infants: Findings from the PARIS birth cohort. Clin. Exp. Allergy.

[33] Liu F., Zhao Y., Liu Y.Q., Liu Y., Sun J., Huang M.M., Liu Y., Dong G.H. (2014). Asthma and asthma related symptoms in 23,326 Chinese children in relation to indoor and outdoor environmental factors: The Seven Northeastern Cities (SNEC) Study. Sci. Total Environ..

[34] Tsai C.H., Tung K.Y., Su M.W., Chiang B.L., Chew F.T., Kuo N.W., Lee Y.L. (2013). Interleukin-13 genetic variants, household carpet use and childhood Asthma. PLoS ONE.

[35] Zock J.P., Jarvis D., Luczynska C., Sunyer J., Burney P., European Community Respiratory Health Survey (2002). Housing characteristics, reported mold exposure, and asthma in the European Community Respiratory Health Survey. J. Allergy Clin. Immunol..

[36] Behrens T., Maziak W., Weiland S.K., Rzehak P., Siebert E., Keil U. (2005). Symptoms of asthma and the home environment. The ISAAC I and III cross-sectional surveys in Münster, Germany. Int. Arch. Allergy Immunol..

[37] Skorge T.D., Eagan T.M., Eide G.E., Gulsvik A., Bakke P.S. (2005). Indoor exposures and respiratory symptoms in a Norwegian community sample. Thorax.

[38] Mommers M., Jongmans-Liedekerken A.W., Derkx R., Dott W., Mertens P., van Schayck C.P., Steup A., Swaen G.M., Ziemer B., Weishoff-Houben M. (2005). Indoor environment and respiratory symptoms in children living in the Dutch-German borderland. Int. J. Hyg. Environ. Health.

[39] Al-Zahrani J.M., Ahmad A., AL-Harbi A., Khan A.M., Al-Bader B., Baharoon S., Shememeri A.A., AL-Jahdali H. (2015). Factors associated with poor asthma control in the outpatient clinic setting. Ann. Thorac. Med..

[40] Voûte P.D., Zock J.P., Brunekreef B., de Jongste J.C. (1994). Peak-flow variability in asthmatic children is not related to wall-to-wall carpeting on classroom floors. Allergy.

[41] Jaakkola J.J., Parise H., Kislitsin V., Lebedeva N.I., Spengler J.D. (2004). Asthma, wheezing, and allergies in Russian schoolchildren in relation to new surface materials in the home. Am. J. Public Health.

[42] Herberth G., Gubelt R., Röder S., Krämer U., Schins R.P., Diez U., Borte M., Heinrich J., Wichmann H.E., Herbarth O. (2009). LISAplus study group. Increase of inflammatory markers after indoor renovation activities: The LISA birth cohort study. Pediatr. Allergy Immunol..

[43] Ebbehøj N.E., Agner T., Zimerson E., Bruze M. (2015). Outbreak of eczema and rhinitis in a group of office workers in Greenland. Int. J. Circumpolar Health.

[44] Allermann L., Wilkins C.K., Madsen A.M. (2006). Inflammatory potency of dust from the indoor environment and correlation to content of NAGase and fungi. Toxicol. In Vitro.

[45] Shu H., Jönsson B.A., Larsson M., Nånberg E., Bornehag C.G. (2014). PVC flooring at home and development of asthma among young children in Sweden, a 10-year follow-up. Indoor Air.

[46] Bean B., Moore B.M., Peterson L.R., Gerding D.N., Balfour H.H. (1982). Survival of influenza viruses on environmental surfaces. J. Infect. Dis..

[47] Tiwari A., Patnayak D.P., Chander Y., Parsad M., Goyal S.M. (2006). Survival of two avian respiratory viruses on porous and nonporous surfaces. Avian Dis..

[48] Cheesbrough J.S., Green J., Gallimore C.I., Wright P.A., Brown D.W.G. (2000). Widespread environmental contamination with Norwalk-like viruses (NLV) detected in a prolonged hotel outbreak of gastroenteritis. Epidemiol. Infect..

[49] Figley D.A., Makohon J.T., Fugler D. The efficiency of clean-up techniques for removing lead contaminated construction dust from floor coverings. Proceedings of the 6th International Conference on Indoor Air Quality and Climate.

